# *MagStREXS*, a crystallographic computer program to determine magnetic structures through resonant elastic X-ray scattering data. I. Fundamental equations

**DOI:** 10.1107/S1600576725006727

**Published:** 2025-09-04

**Authors:** Pablo J. Bereciartua, Juan Rodríguez-Carvajal, Sonia Francoual

**Affiliations:** a Deutsches Elektronen-Synchrotron DESY Hamburg Germany; b Institut Laue–Langevin (ILL), Grenoble, France; Oak Ridge National Laboratory, USA

**Keywords:** resonant X-ray magnetic scattering, REXS, magnetic structures, computer programs

## Abstract

Resonant elastic X-ray scattering (REXS) is an experimental technique that can be highly valuable for studying magnetic structures in certain scenarios. In this paper, we introduce *MagStREXS*, a crystallographic program designed to determine magnetic structures from REXS data. This software makes use of the key concepts and computational tools already established in the field of magnetic crystallography. Here, we present the fundamental equations implemented in *MagStREXS*, derived for the two methods used to describe magnetic models: the representation analysis and the symmetry-based approach. We illustrate the capabilities of *MagStREXS* with a case study. The software is under active development, with the aim of benefiting both specialists and non-specialists in the field.

## Abstract 2

This article presents the fundamental concepts and equations implemented in the program *MagStREXS* to determine magnetic structures based on resonant elastic X-ray scattering diffraction data.

## 1. Introduction

The determination of magnetic structures is crucial not only for identifying functional materials with new technological applications but also for understanding underlying phenomena in complex compounds. While some magnetic structures, such as ferromagnets, are relatively simple, others can be highly intricate (*e.g.* incommensurate phases, multi-**k** structures or magnetic structures involving propagation vectors from different **k**-stars), making the study of magnetic structures a challenging area of research in solid-state physics (Rodríguez-Carvajal & Villain, 2019 ▸).

In many cases, magnetic structures are described using the widely adopted representation analysis approach. However, the complete characterization of a magnetic phase requires the identification of its symmetry (Perez-Mato *et al.*, 2015 ▸). Symmetry governs many properties of magnetic compounds, such as phase transitions, domain formation, various phenomena in multiferroics (Perez-Mato & Ribeiro, 2011 ▸) and specific topologies of electronic wavefunctions (Bernevig *et al.*, 2022 ▸). Furthermore, symmetry is instrumental in determining magnetic structures: the list of magnetic models compatible with a given atomic structure can be established from symmetry considerations and the calculation of magnetic structure factors can be simplified using symmetry operations, enhancing the efficiency of the refinement of magnetic structures (Petříček *et al.*, 2010 ▸).

Due to the complexity of many magnetic structures, their accurate description and analysis in terms of either irreducible representations (irreps) or magnetic space/superspace groups (MSGs/MSSGs) are only possible thanks to dedicated crystallographic databases and computational tools [see the lists provided by Perez-Mato *et al.* (2015 ▸) or Rodríguez-Carvajal & Villain (2019 ▸)]. Over the past fifty years, advances in the systematic description of magnetic structures have progressed alongside improvements in neutron diffraction, making this technique the predominant method for studying magnetism (Damay, 2015 ▸; Dronskowski *et al.*, 2024 ▸). Crystallographic tools for studying magnetic phases have thus been developed in tandem with programs for analysing neutron diffraction data, integrating them to facilitate magnetic structure determination. Today, neutron powder diffraction (NPD) is the most common approach for magnetic structure studies, although it has limitations and complementary techniques such as single-crystal diffraction or polarized neutron experiments are often required to characterize a magnetic structure fully.

Resonant elastic X-ray scattering (REXS) is a powerful synchrotron-specific technique employed to probe charge, spin and orbital long-range orderings (Matteo, 2012 ▸). It is widely used as a complementary technique to NPD for magnetic structure determination in situations where single-crystal neutron diffraction is not feasible, *i.e.* in the following scenarios: (i) when the magnetic ions in the material have a high neutron absorption cross section (*e.g.* Gd, Eu, Ir *etc.*), (ii) when sufficiently large single crystals cannot be grown, (iii) when two or more magnetic structures cannot be distinguished using available neutron diffraction data, which is a particular challenge in NPD (Shirane, 1959 ▸; Rodríguez-Carvajal & Bourée, 2012 ▸), (iv) when the highest resolution of X-rays is required, and/or (v) when multiple magnetic ions are present. Indeed, REXS offers the unique advantage of being element- and shell-specific, whereas non-resonant elastic X-ray scattering and neutron diffraction measure the total magnetization.

However, REXS is significantly hindered by the lack of dedicated magnetic crystallographic software for analysing this type of data, which prevents the technique from reaching its full potential. Researchers conducting REXS experiments are often forced to perform calculations manually or using simple computer scripts, despite the inherent complexity of both magnetic structures and the REXS scattering cross sections. This can lead to inaccuracies and considerable delays in data analysis.

In this paper, we present the foundational principles behind *MagStREXS*, a crystallographic program currently under active development to address this gap and provide a dedicated tool for analysing magnetic structures using REXS diffraction data. The program is built upon the theoretical concepts established in crystallography for the description and analysis of magnetic structures. As such, the current version of *MagStREXS* can accommodate any model constructed within the representation analysis approach, commensurate or incommensurate, as well as any model based on an MSG. The use of MSSGs for describing incommensurate magnetic phases, while planned, is not yet fully implemented, so we will not elaborate on this specific case here.

The functionalities currently implemented in *MagStREXS* focus on two types of data: the integrated intensities of a set of magnetic reflections, and the Poincaré–Stokes parameters as measured in a full linear polarization analysis (FLPA). The analysis of azimuthal dependence data, which is also important, is in the process of being implemented and will be discussed in the near future. *MagStREXS* is currently available as a beta version on the computing platform of DESY, and users can employ it at present to analyse data collected on beamline P09 at PETRA III.

In this paper, we focus on discussing the fundamental equations incorporated into *MagStREXS* and present a case study of a magnetic structure determination as a proof of concept. A comprehensive description of *MagStREXS* and its application to the characterization of magnetic structures will be provided in a separate paper once a distributable version of the program has been finalized.

## 2. Magnetic crystallography

Magnetic crystallography provides a systematic framework for describing and studying magnetic structures, which is highly beneficial for the type of analysis conducted within *MagStREXS*. In this section, some essential concepts regarding the crystallographic description of such structures are briefly introduced, while more comprehensive discussions of this topic can be found elsewhere (Rodríguez-Carvajal & Bourée, 2012 ▸; Rodríguez-Carvajal & Villain, 2019 ▸; Perez-Mato *et al.*, 2015 ▸; Perez-Mato *et al.*, 2012 ▸; Petříček *et al.*, 2010 ▸).

Historically, two distinct approaches for describing magnetic structures have been developed within the crystallographic context. One of these, known as representation analysis, is based on the application of group theory to crystallographic space groups, while the other is founded on the symmetry associated with the ordering of magnetic moments in a crystal. Each approach offers its own systematic method for identifying and describing all magnetic structures compatible with a given crystal structure in which some atoms possess magnetic moments.

Developed in parallel, these two methodologies were initially considered alternatives or even mutually exclusive. However, this debate has finally been resolved, and it has been demonstrated that combining the two approaches provides the most complete and effective means of describing and understanding magnetic structures (Rodriguez-Carvajal & Perez-Mato, 2024 ▸). This complementary relationship is the reason why any software dedicated to the study of magnetic structures should incorporate the capability to analyse magnetic models based on either of these two descriptions.

Among the various computational tools mentioned in the references given in the *Introduction*[Sec sec1], one has been particularly crucial for the development of *MagStREXS*: the *Crystallographic Fortran Modules Library* (*CrysFML*). This library is a collection of Fortran modules designed to simplify the development of crystallographic software (Rodríguez-Carvajal & González-Platas, 2002 ▸; Rodríguez-Carvajal & González-Platas, 2023 ▸). While there are other crystallographic libraries, such as the *Cambridge Crystallographic Subroutine Library* (Matthewman *et al.*, 1982 ▸) and the *Computational Crystallography Toolbox* (Grosse-Kunstleve *et al.*, 2002 ▸), *CrysFML* was the only suitable option for creating *MagStREXS*. Firstly, this library benefits from the efficiency and simplicity of modern Fortran. Secondly, *CrysFML* defines numerous derived types (data structures) that are well suited to describing the various elements required for the analysis performed with *MagStREXS*, such as atomic and magnetic structures, the symmetry of these models, and the data collected in diffraction experiments. Finally, the library includes a wide range of functionalities, from general-purpose procedures, such as mathematical functions and subroutines for string manipulation, to more specific crystallographic computing procedures, such as those for handling symmetry, sets of reflections, and atomic and magnetic structures. The combination of these features has greatly facilitated the development of *MagStREXS*.

While the original version of *CrysFML* was written in Fortran 90/95, it has now been superseded by *CrysFML2008*, a new version based on the latest Fortran standard (Fortran 2023 at the time of writing) (Rodríguez-Carvajal & González-Platas, 2025 ▸). This updated version exploits new language features, such as submodules and object-oriented capabilities, enabling the implementation of a symmetry-based description of magnetic structures in *MagStREXS*.

### 2.1. Description based on representation analysis

According to Landau theory, representation analysis assumes that magnetic orderings arising from a phase transition can be described using the basis functions corresponding to a single irrep of the magnetic representation associated with the parent structure (Bertaut, 1968 ▸; Bertaut, 1981 ▸; Izyumov & Naish, 1979 ▸; Izyumov *et al.*, 1979*b* ▸; Izyumov *et al.*, 1979*a* ▸). In this framework, any possible magnetic ordering is represented by a Fourier series,
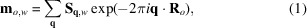
where **m**_*o*,*w*_ is the magnetic moment of atom *w* in the unit cell associated with the lattice vector **R**_*o*_ (the position vector of the origin of cell *o*), with the atom located at **R**_*o*,*w*_ = **R**_*o*_ + **r**_*w*_. The use of a minus sign in the exponential factor is discussed in Appendix *A*[App appa]. The set of {**q**} vectors represents the propagation vectors involved in the description of the magnetic structure. These vectors are determined experimentally and define the relationship between magnetic moments located in different unit cells of the atomic structure. The Fourier coefficients **S**_**q**,*w*_ are, in general, complex vectors and must satisfy 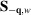 = 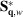 to ensure that the magnetic moment **m**_*q*,*w*_ is a real vector. These Fourier coefficients **S**_**q**,*w*_ can be expressed as a linear combination of the basis functions of the irrep used to construct the magnetic model, 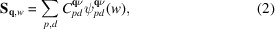
where ν is the label of the active irrep, *d* is an index related to the dimension of this irrep and *p* indicates the number of times the irrep ν appears in the decomposition of the magnetic representation. The basis functions are represented by the vectors 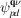. The parameters 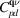 are the mixing coefficients used when a magnetic model involves a combination of multiple basis functions. If the description of the magnetic structure includes more than one irrep, a sum over ν should also be included in equation (2).

One advantage of the representation analysis approach is its ability to describe both commensurate and incommensurate phases, as equation (1) is applicable in both cases, and the components of the propagation vector(s) can be either rational or irrational. However, a limitation of this approach is that it does not incorporate information about the invariance symmetry of the magnetic structure.

### 2.2. Description based on symmetry

In the symmetry-based approach for describing magnetic ordering, the set of magnetic models compatible with a given atomic structure is identified and classified according to their symmetry. This description requires distinguishing between commensurate and incommensurate structures, as the symmetry of the former is described by an MSG, also known as a Shubnikov group, while the symmetry of the latter can only be characterized using an MSSG. To describe a magnetic structure within this framework, it is necessary to define its magnetic unit cell, the smallest building block whose periodic repetition reproduces the magnetic structure. This magnetic unit cell is defined by the vectors {**a**_mag_, **b**_mag_, **c**_mag_} and is generally different from the unit cell used to describe the atomic structure, which is defined by the vectors {**a**, **b**, **c**}. Other essential components of this description include the list of atomic positions {**r**_*w*_} and the symmetrically independent magnetic moments. In the context of the REXS technique, the magnetic moments are replaced by unit vectors {**z**_*w*_} representing the orientation of these moments.

The symmetry group that leaves a magnetic ordering invariant is characterized by the coset representatives with respect to the translation group. This set of symmetry operations allows the enumeration of atoms and magnetic moments that are symmetrically equivalent within a magnetic unit cell. It is useful to describe the action of a symmetry operation on atomic positions and magnetic moments, as this is employed in the calculation of the structure factor detailed in Section 3. Using the Seitz notation, a generic symmetry operation 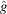 within an MSG can be written as 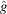 = {*g*, δ|**t**}, where *g*, δ and **t** represent the rotational part, the signature and the translational part of the symmetry operator, respectively. The signature δ is related to time reversal (denoted by the symbol 1′), an operation that acts exclusively on magnetic moments according to 1′**m** = −**m**. If time reversal is part of the symmetry operation 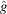, the value of δ is −1; otherwise, it is 1. With this notation, the action of a symmetry operation on the position and magnetic moment of an atom can be expressed, respectively, as
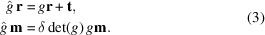
A more detailed discussion of symmetry operators within an MSG and their action on various atomic features can be found in other references (Rodríguez-Carvajal & Villain, 2019 ▸; Petříček *et al.*, 2010 ▸).

## 3. REXS structure factor for electric dipole transitions

According to the kinematical approximation, the intensity of a diffracted X-ray beam is proportional to |*F*|^2^, where *F* is the structure factor of the whole crystal. This structure factor can be expressed as
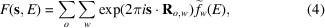
where the summations over *o* and *w* include all unit cells of the crystal and all atoms within the unit cell, respectively. Note that, to facilitate comparison with similar calculations found in other references (Rodríguez-Carvajal & Villain, 2019 ▸; Petříček *et al.*, 2010 ▸), we use the crystallographic convention, where 2π is explicitly written in the exponential factor, the scattering vector **Q** = **k**_f_ − **k**_i_ = 2π**s** is expressed in terms of the vector **s**, and **H** represents a vector of the reciprocal lattice.

The atomic scattering factor can be expressed as
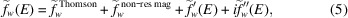
where the last two terms become significant near an absorption edge. The term 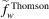 corresponds to the Thomson scattering factor, given by
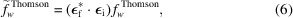
where **ε**_i_ and **ε**_f_ are the polarization vectors of the incident and diffracted beams, respectively. The term 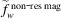 represents the non-resonant magnetic scattering factor (Blume & Gibbs, 1988 ▸), which is currently not computed in *MagStREXS*. An enumeration of the different multipole terms that can appear in 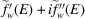 is provided by Matteo (2012 ▸) and Matsumura (2017 ▸), along with references therein. In *MagStREXS* at present, we only retain the electric dipole term (Hill & McMorrow, 1996 ▸; Altarelli, 2006 ▸), expressed as
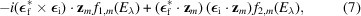
where **z**_*m*_ is the unit vector in the direction of the magnetic moment of the *m*th ion of the atom type λ. The factors *f*_1,*m*_(*E*_λ_) and *f*_2,*m*_(*E*_λ_) are two scalars related to the strength of the resonant process at the energy *E*_λ_ at which the experiment is performed.

In the following subsections, the expression of the structure factor is calculated within the two different approaches used to describe magnetic structures (see Section 2). Obviously, a magnetic structure is independent of the approach used to describe it, and accordingly, REXS structure factors calculated using different approaches must be consistent, as shown in the case study presented in Section 6.

### 3.1. REXS structure factor for electric dipole transitions within the representation analysis approach

In the representation analysis approach, the vectors **z**_*o*,*m*_ can be calculated as a Fourier series similar to the one given in equation (1).

By introducing equations (5), (6), (7) and (1) into equation (4), and considering the assumptions outlined above, the structure factor can be expressed as
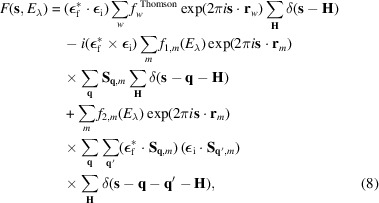
where the summation index *m* runs only over the magnetic atoms of type λ in the unit cell, and the factors *f*_1,*m*_(*E*_λ_) and *f*_2,*m*_(*E*_λ_) may vary between symmetrically independent sites. The Fourier coefficients **S**_**q**,*m*_ are analogous to those in equation (1). The double summation over **q** and **q**′ in the third term accounts for all combinations that can arise in a magnetic structure with more than one propagation vector. The summations over **H** include all vectors of the reciprocal lattice. The Dirac delta functions in equation (8) represent the diffraction conditions, determining the contribution of each term at different positions in reciprocal space. The first term contributes to charge Bragg reflections (with no dependence on the magnetic structure), while the second and third terms give rise to first- and second-order magnetic reflections, respectively.

The expression in equation (8) is valid for all kinds of magnetic structures, commensurate and incommensurate, including those with multiple propagation vectors. It is useful to rearrange the terms to highlight the contributions associated with different magnetic models. For example, in a magnetic structure with more than one propagation vector, the vectors **q** and **q**′ can be equal to or different from **0**, meaning the third term in equation (8) can be reorganized as 
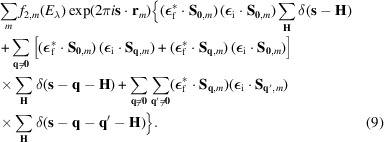
Here, terms with **q** = **0** or **q**′ = **0** are explicitly indicated (note the Fourier coefficients **S**_**0**,*m*_). This rearrangement shows that the Dirac delta functions δ(**s** − **H**), δ(**s** − **q** − **H**) and δ(**s** − **q** − **q**′ − **H**) produce diffracted intensities at different positions in reciprocal space, with two exceptions. The first occurs in magnetic structures involving a propagation vector that is half of a reciprocal-lattice vector (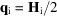), with the combination **q** = **q**′ = **q**_i_. The second arises in incommensurate phases with the combination **q** = **q**_i_ and **q**′ = −**q**_i_, as the mathematical description of these structures always involves the pair (**q**_i_, −**q**_i_) for propagation vectors with one (or more) irrational components. In both cases, the contribution of the third term in equation (9) overlaps in reciprocal space with contributions associated with the Dirac delta function δ(**s** − **H**).

To clarify equations (8) and (9), it is helpful to write the REXS structure factor explicitly at different positions in reciprocal space. For a particular reflection at position **s**_0_ = **H**_0_, where **H**_0_ is a vector of the reciprocal lattice associated with the atomic unit cell, the structure factor is
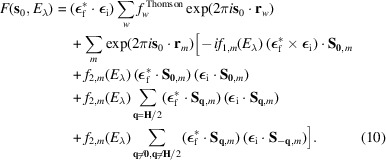
Here, the summation over *w* is relevant only when the reflection **H**_0_ satisfies the reflection conditions of the space group of the atomic structure. The first and second terms in the summation over *m* are included only if **q** = **0** is one of the propagation vectors describing the magnetic structure. The summation of the third term runs over propagation vectors of the form 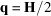, and the summation of the last term runs over propagation vectors that comply the conditions **q** ≠ **0** and 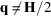.

For a first-order magnetic reflection at position **s**_1_ = **H**_0_ + **q**_1_, the structure factor is
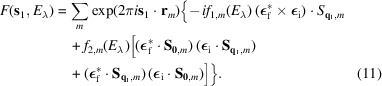
The second term in equation (11) is relevant only for magnetic structures with more than one propagation vector, when the vector **q** = **0** is one of them (*e.g.* an antiferromagnetic structure with a ferromagnetic component).

Finally, for a second-order magnetic reflection at position **s**_2_ = **H**_0_ + **q**_1_ + **q**_2_, the structure factor is
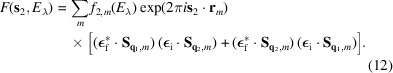
Here, it is assumed that **q**_1_ and **q**_2_ are different from **0**, **q**_2_ ≠ −**q**_1_ and **q**_1_ + **q**_2_ ≠ **H**.

### 3.2. REXS structure factor for electric dipole transitions within the symmetry-based approach for the commensurate case

In the symmetry-based approach for describing magnetic ordering, it is necessary to distinguish between commensurate and incommensurate structures. Since the description of incommensurate magnetic phases using MSSGs is not yet fully implemented in *MagStREXS*, we focus here on the REXS structure factor for commensurate magnetic phases.

By considering equations (4), (5), (6) and (7), along with the assumptions discussed in Section 3, the REXS structure factor for a commensurate magnetic phase can be expressed as
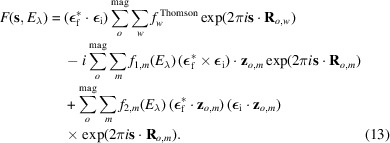
Here, the summations over *o*, *w* and *m* run over all magnetic unit cells (mag) of the structure, all atoms in one magnetic unit cell and all magnetic atoms of type λ in one magnetic unit cell, respectively. This structure factor can be further decomposed into
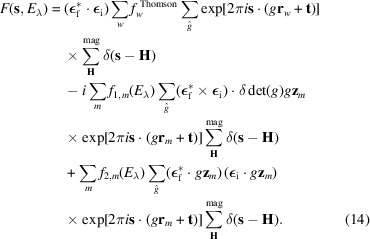
In this expression, the summations over *o* and *m* have been rearranged to run only over the symmetrically independent atoms and magnetic atoms of type λ, respectively. The summation over 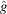 spans the symmetry operations associated with each Wyckoff position. The action of these symmetry operators on the positions and magnetic moments of the atoms has been explicitly indicated (see Section 2 for further details). Note that a factor [δdet(*g*)]^2^ = 1 has been omitted in the third term. The Dirac delta functions δ(**s** − **H**) give rise to diffracted intensities at positions corresponding to reciprocal lattice vectors associated with the magnetic unit cell.

The final expression for the structure factor corresponding to a particular reflection at position **H**_0_, where **H**_0_ is a vector of the reciprocal lattice associated with the magnetic unit cell, is
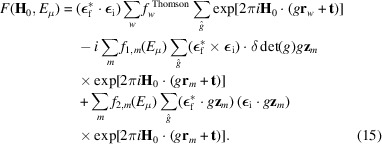
In the symmetry-based description of a commensurate magnetic structure, no propagation vector is used to characterize the distribution of diffracted intensities, as all reflections are associated with a reciprocal lattice vector of the magnetic unit cell. The contributions of each term in equation (15) to different positions in reciprocal space depend not only on the orientation of the magnetic moments but also on the MSG. Similarly to non-magnetic diffraction, certain symmetry operations in the MSG of the magnetic structure give rise to systematic absences, as discussed for magnetic non-polarized neutron diffraction by Li (1956 ▸) and Gallego *et al.* (2012 ▸). A complete discussion of the possible contributions and reflection conditions (or extinction rules) associated with the different terms in this equation is beyond the scope of this article.

## 4. Density matrix formalism

REXS experiments can be conducted in various diffraction geometries, with different polarizations of the incident X-ray beam and in different polarization channels. For example, in an FLPA, the Poincaré–Stokes (P-S) parameters of the diffracted beam are typically measured in a fixed diffraction geometry, while the direction of the electric field vector of the linearly polarized incident beam is rotated around **k**_i_, the wavevector associated with the incoming photons. To account for all these possibilities, the density matrix formalism must be employed. For instance, within this formalism, the intensity (*I*) and the P-S parameters (*P*_1_, *P*_2_, *P*_3_) of a given X-ray beam can be calculated as
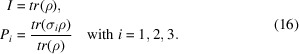
Here, ρ is a matrix representing the state of the X-ray beam, which can be completely or partially polarized, and the matrices 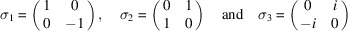
are the Pauli matrices. A more detailed discussion of this formalism can be found in the articles by Blume & Gibbs (1988 ▸) and Detlefs *et al.* (2012 ▸), and references therein.

Within this formalism, the effect of the different elements interacting with the X-ray beam (such as the sample, a phase retarder or an analyser) is calculated by combining the density matrix describing the state of the X-ray beam with the corresponding Jones matrix using the expression
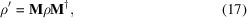
where ρ and ρ′ are the density matrices associated with the incident and scattered beam, respectively, **M** is the Jones matrix of the element interacting with the X-ray beam, and **M**^†^ is the conjugate transpose of **M**. Thus, the combined effect of the multiple scatterers on the X-ray beam can be determined by successively applying equation (17) with the Jones matrix associated with each element in the beam path.

The polarization state of the incident beam is characterized by the P-S parameters *P*_1_, *P*_2_ and *P*_3_. These parameters can be combined to calculate the density matrix of the incident beam, given by
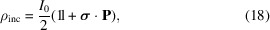
where the matrices σ_1_, σ_2_ and σ_3_ are the Pauli matrices mentioned above.

The Jones matrix for the sample is written as a 2 × 2 matrix, using **ε**_σ_ and **ε**_π_ as basis vectors to describe the different polarization states involved in the scattering process:
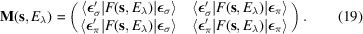
Here, each element of the matrix corresponds to one of the REXS structure factors calculated in Section 3, and the initial and final polarization states have been replaced by the basis vectors **ε**_σ_ and **ε**_π_. Note that the Jones matrix depends on the position in reciprocal space for which the scattering contributions are being calculated, as specified by the vector **s**. Finally, the intensities and P-S parameters are calculated by combining equation (19) with equations (17) and (16).

## 5. Agreement factors

The type of analysis conducted with *MagStREXS* involves evaluating different magnetic models to account for the experimental data. In addition to the graphical representations typically used in such analyses to compare calculated and measured values, several agreement factors have been computed to assess the quality of the fit for each examined model.

For datasets consisting of a set of integrated intensities, the Bragg factor *R*_B_ is used as an agreement factor. This factor is a standard criterion in crystallography for analysing single-crystal diffraction data. *R*_B_ is calculated using the expression 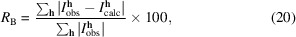
where 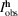 and 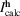 represent the experimental and calculated values, respectively, of the integrated intensity associated with reflection **h**, and the summation includes all reflections in the dataset.

To estimate the agreement between the observed and calculated values for FLPA data, an *R* factor adapted to the P-S parameters *P*_1_ and *P*_2_ has been employed. This factor, denoted by *R*_PS_, is given by the expression
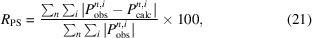
where the summation over *n* includes the different points associated with the set of values of the polarization angle η selected during FLPA data collection, and the index *i* = 1, 2 corresponds to the P-S parameters *P*_1_ and *P*_2_, respectively.

## 6. Case study: antiferromagnetic phase of compound EuNi_2_Ge_2_

To demonstrate the capabilities of *MagStREXS*, we selected a material with a well known commensurate magnetic structure. The compound EuNi_2_Ge_2_ was reported to undergo an antiferromagnetic phase transition at *T*_N_ = 30.8 K, featuring magnetic reflections associated with the propagation vector **q** = (0, 0, 1). Due to the large neutron absorption cross section of Eu, the magnetic phase of this compound was previously investigated using REXS. It was concluded that the magnetic moments lie in the *ab* plane of the tetragonal structure, on the basis of the dependence of the integrated intensities of the measured reflections on |**Q**| (Islam *et al.*, 1999 ▸). Given the limited data available for this compound and the fact that FLPA in REXS was only proposed several years after that initial study (Mazzoli *et al.*, 2007 ▸; Johnson *et al.*, 2008 ▸; Scagnoli *et al.*, 2009 ▸), a new REXS experiment was conducted on the P09 beamline at PETRA III (DESY). Data were collected at 5 K, with the energy of the incident beam tuned to the Eu *L*_II_ absorption edge (7614 eV). Exploiting the experimental capabilities available on the P09 beamline (Strempfer *et al.*, 2013 ▸; Francoual *et al.*, 2013 ▸), two types of data were collected: the integrated intensities of a set of ten magnetic reflections measured in the σπ′ channel, and FLPA datasets for the magnetic reflections 005, 009 and 016.

### 6.1. Analysis based on representation analysis

In this approach, the list of models that can describe the magnetic structure of the compound EuNi_2_Ge_2_ is generated through representation analysis performed using the software *BasIreps* [a program within the *FullProf* suite (Rodríguez-Carvajal, 1993 ▸)]. The information required for this analysis includes the space group of the atomic structure (*I*4/*mmm*, No. 139), the position of the Eu atom [located at **r** = (0, 0, 0), Wyckoff site 2*a*] and the propagation vector [**q** = (0, 0, 1)]. In this case, the decomposition of the magnetic representation involves two irreps,
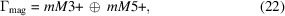
where *mM*3+ and *mM*5+ are one- and two-dimensional irreps, respectively. The basis vectors associated with each irrep are given in Table 1 ▸. The magnetic model based on *mM*3+ involves only one basis function, which determines that the magnetic moments are oriented along the *c* axis, while the most general model based on irrep *mM*5+ is a linear combination of basis functions ψ_2_ and ψ_3_, with the magnetic moments lying in the *ab* plane.

Four different models were examined with *MagStREXS*: models 1, 2 and 3 correspond to the magnetic orderings associated with basis functions ψ_1_, ψ_2_ and ψ_3_, respectively, while model 4 is constructed as a combination of basis vectors ψ_2_ and ψ_3_. In this last model, two mixing coefficients (*C*_2_ and *C*_3_) are included to combine the two basis functions involved [see equation (2) for more details]. These coefficients are the free parameters of the model that determine the specific orientation of the magnetic moments within the *ab* plane, and *MagStREXS* can be used to find the values corresponding to the best fit, since a refinement procedure is implemented in the current version of the software.

**Figure 1:**
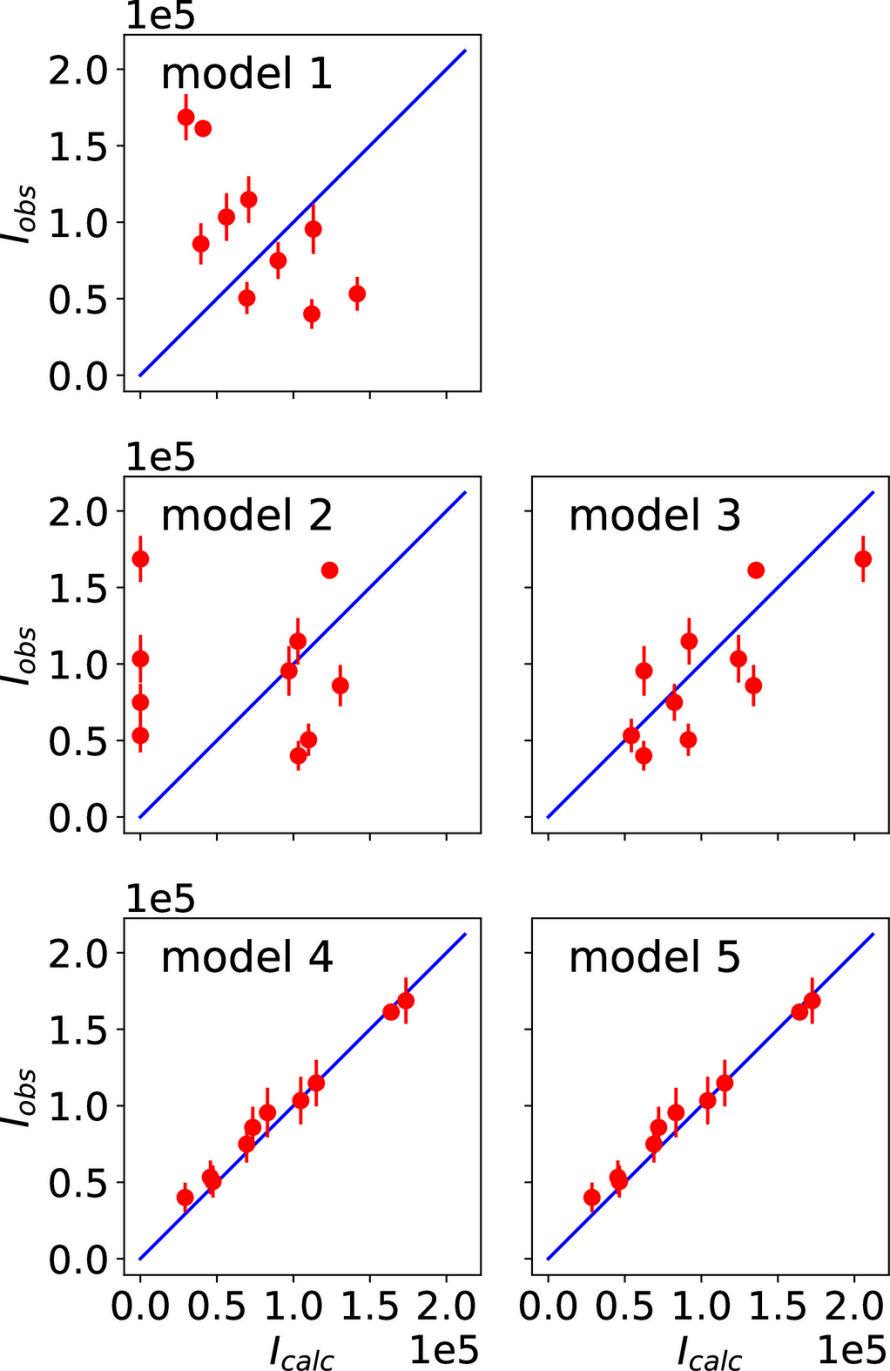
Comparison of observed and calculated integrated intensities for the magnetic models considered within the representation analysis approach. The blue lines represent the ideal case where the observed and calculated intensities are equal. The values calculated for models 4 and 5 provide the best fit to the distribution of intensities measured experimentally. The figures obtained for these two models are essentially the same.

**Figure 2:**
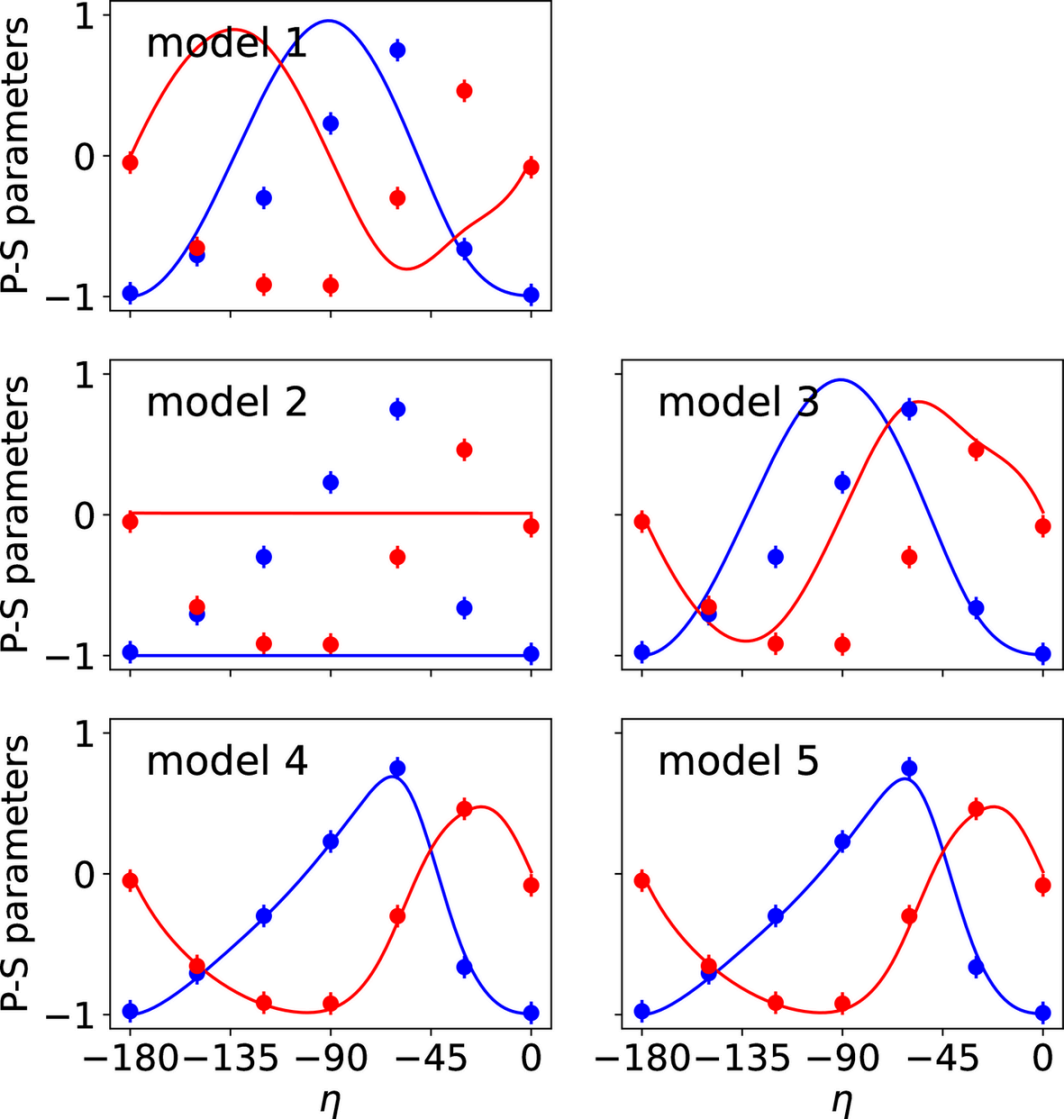
Observed and calculated P-S parameters *P*_1_ (blue) and *P*_2_ (red) corresponding to the reflection 005 for the different magnetic models. The figures represent the variation in P-S parameters with respect to the angle of polarization of the incident X-ray beam (denoted η). The coloured circles and continuous lines represent the observed and calculated values, respectively. Only the values calculated for models 4 and 5 reproduce the P-S parameters measured experimentally. The figures obtained for these two models are essentially the same.

The two types of data collected in the REXS diffraction experiment were used simultaneously in the fits performed with *MagStREXS* for each of the magnetic models. The calculated intensities corresponding to each of the four magnetic models are compared with the experimental values in Fig. 1 ▸, while a comparison between the measured and calculated P-S parameters is shown in Fig. 2 ▸. Additional figures displaying the P-S parameters corresponding to reflections 009 and 016 are provided in Appendix *B*[App appb]. The agreement factors obtained for each magnetic model and experimental dataset are given in Table 2 ▸ (see Section 5 for more details). The results from *MagStREXS* for each of the examined models show that the best fit to the experimental data corresponds to model 4. The magnetic structure is ordered according to irrep *mM*5+, with magnetic moments lying in the *ab* plane. The values of the mixing coefficients corresponding to the best fit obtained in the refinement procedure carried out with model 4 are
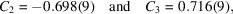
where *C*_2_ and *C*_3_ are the mixing coefficients associated with basis functions ψ_2_ and ψ_3_, respectively. Considering the basis functions used to construct the magnetic model (Table 1 ▸) and how they are combined with the mixing coefficients to calculate the magnetic moments [equations (1) and (2)], the obtained values indicate that the magnetic moments are almost parallel to the direction [1
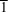
0]. In this compound, this direction is special because the space group of the atomic structure contains a twofold axis along this direction, which is part of the site symmetry of the position occupied by the Eu atom. This can have implications for the symmetry of the magnetic structure, and therefore it is convenient to consider a new model with the magnetic moments restricted to the direction [1
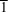
0]. Model 5 introduces such a constraint by using the same combination of basis functions ψ_2_ and ψ_3_ as in model 4 but fixing the mixing coefficients to the values 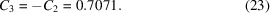


**Figure 3:**
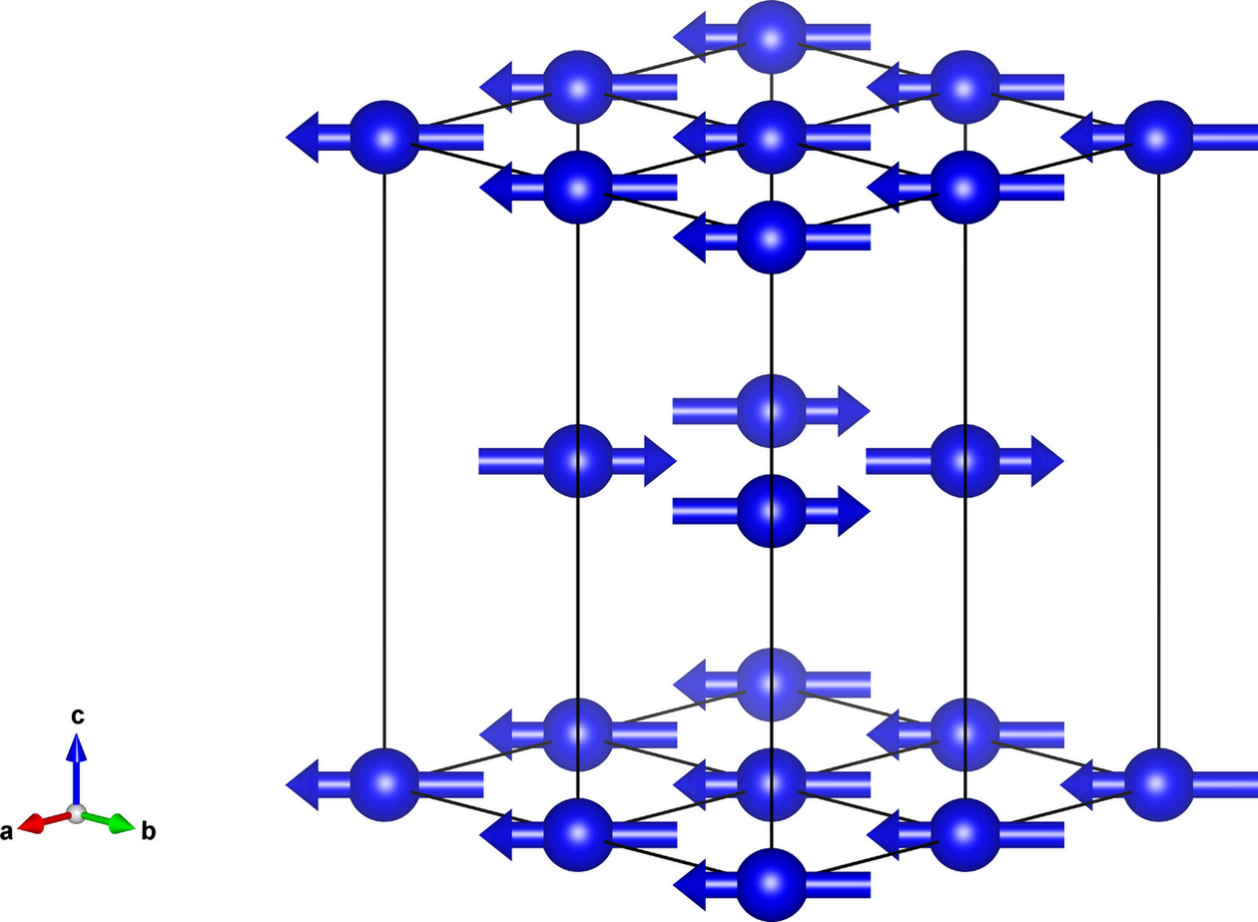
Magnetic structure of EuNi_2_Ge_2_ shown in four adjacent unit cells. In this case, the atomic and magnetic structures can be described with the same unit cell. The magnetic moment distribution is ferromagnetic within *ab* planes and antiferromagnetic along the *c* axis.

As with the previously examined models, the intensities and P-S parameters corresponding to model 5 were calculated with *MagStREXS* and compared with the experimental values. The figures obtained with both types of data are nearly identical to those obtained for model 4. The agreement factors calculated for the two models are also similar (see Table 2 ▸), with the agreement factors of model 5 slightly higher but not enough to discard this model in favour of model 4. The magnetic structure corresponding to model 5 is shown in Fig. 3 ▸.

Although the results obtained with models 4 and 5 are close, the two models are conceptually different. In model 4, the magnetic moments can be oriented along any direction in the *ab* plane, while model 5 is more restrictive, forcing the magnetic moments to align along [1
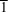
0]. Since these restrictions are usually related to the symmetry of the magnetic structure, it is clear that model 5 corresponds to a magnetic structure with higher symmetry. To clarify this point, it is convenient to perform the analysis of the magnetic structure within the symmetry-based approach, which is the focus of the next subsection.

### 6.2. Analysis based on symmetry

In the symmetry-based approach, the list of models to be considered for describing the magnetic structure of the compound EuNi_2_Ge_2_ is generated using the tool *MAXMAGN* from the Bilbao Crystallographic Server (Perez-Mato *et al.*, 2015 ▸). This program identifies all possible maximal magnetic symmetries compatible with the symmetry of the atomic structure (*I*4/*mmm*, No. 139) and the experimentally determined propagation vector [**q** = (0, 0, 1)]. It then derives the models compatible with each of these symmetries. If the position of the magnetic atom [Eu atom, located at **r** = (0, 0, 0)] is also provided, *MAXMAGN* can determine the possible magnetic moment orientations for each model on the basis of symmetry constraints associated with each atomic position. The list of magnetic models obtained from this tool is given in Table 3 ▸, including the MSG associated with each model. Note that the symbol and number of each MSG provided in this table and throughout this paper are given in the Belov–Neronova–Smirnova (BNS) system (Belov *et al.*, 1957 ▸). The table also includes the transformation from the basis of the atomic structure to the standard setting of each MSG for each model, indicated using the shorthand notation employed in *International Tables for Crystallography* (Aroyo, 2016 ▸). Note that models 7 and 8 (and, analogously, models 9 and 10) are associated with the same MSG but with different transformations, indicating that the two models contain the same set of symmetry elements but with different orientations relative to the basis of the atomic structure. As a result, the symmetry constraints differ and the magnetic moments are restricted to different directions in each model. Finally, note that almost all models in Table 3 ▸ have a one-to-one correspondence with models in Section 6.1; models 6, 7, 9 and 10 are equivalent to models 1, 5, 3 and 2, respectively.

**Figure 4:**
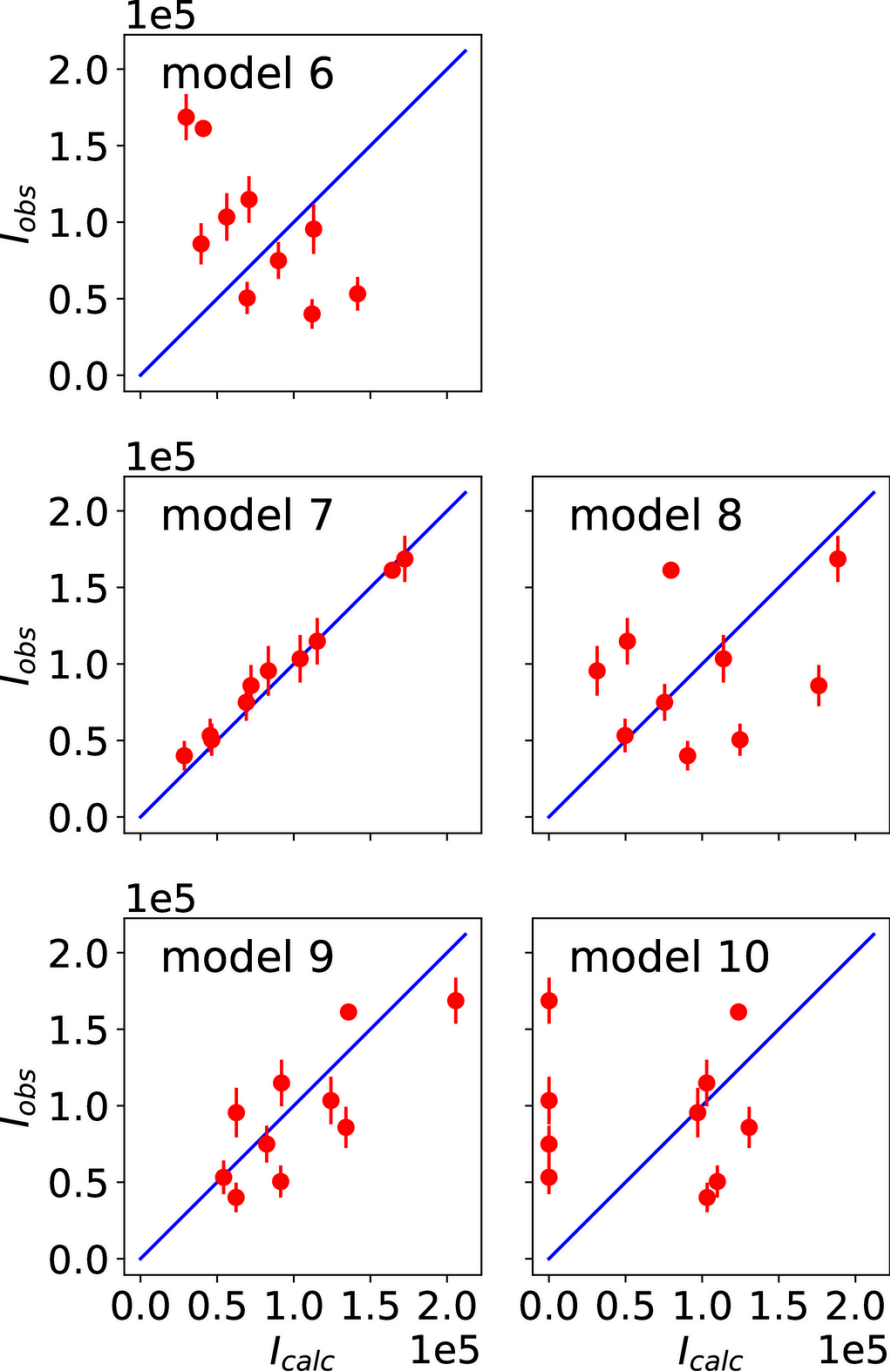
Observed and calculated integrated intensities for the magnetic models listed in Table 3 ▸. The blue lines represent the ideal case where the observed and calculated intensities are equal. The best fit to the experimental intensities corresponds to the intensities calculated for model 7.

The observed and calculated intensities and P-S parameters for the different models are shown in Figs. 4 ▸ and 5 ▸, respectively. Additional figures presenting the P-S parameters corresponding to reflections 009 and 016 are provided in Appendix *B*[App appb]. The agreement factors obtained for each magnetic model are given in Table 4 ▸. Model 7 leads to the best results, confirming that it represents the magnetic structure of the studied compound, as concluded in Section 6.1. Since model 5 and model 7 are the same, the symmetry of model 5 is given by MSG *C*_*A*_*mca* (No. 64.480).

## 7. Conclusions

The purpose of *MagStREXS* is to facilitate the analysis of magnetic structures using resonant elastic X-ray scattering diffraction data, drawing on the concepts and computational tools developed within magnetic crystallography. Symmetry considerations have only recently been incorporated in the analysis of REXS data, primarily limited to the representation analysis approach (Nandi *et al.*, 2011 ▸; Williams *et al.*, 2016 ▸; Donnerer *et al.*, 2016 ▸). Due to the inherent complexity of magnetic structures, their accurate description and analysis require the use of either representation analysis or magnetic space groups/magnetic superspace groups, or ideally, both. In this paper, we have presented the fundamental equations for the REXS structure factor associated with electric dipolar transitions (*E*1–*E*1) as currently implemented in the *MagStREXS* software for these two crystallographic approaches. We have illustrated the results of the analysis obtained with *MagStREXS* for both types of description using real experimental data collected for the commensurate magnetic structure of the compound EuNi_2_Ge_2_.

The use of *MagStREXS* should enable researchers to achieve scientific outcomes promptly, preventing them from being overwhelmed by the intricate details of the calculations. Users might employ *MagStREXS* for a full analysis of their data or to double-check their calculations. In an ideal scenario, users will have determined the magnetic structure of the studied compound by the time their experiment on the beamline is completed. In the case study of the compound EuNi_2_Ge_2_ presented in this paper, the determination of the magnetic structure was relatively straightforward. While preparing the experimental setup and collecting data took three days, the data analysis work took less than five hours, with most of the effort dedicated to data reduction and preparing the files to introduce the different models into *MagStREXS*. Computation times are of the order of tens of seconds, making it possible to explore different magnetic structures in a short time. In other cases, the data analysis is more demanding because the studied phase requires more complex magnetic models than initially considered, and care must be taken in constructing these alternative models. Occasionally, results obtained with *MagStREXS* during a beamtime session might indicate that additional data are required, which is still valuable information for refining the experimental strategy.

*MagStREXS* has been available as a beta version since 2022, with users of beamline P09 having the opportunity to run the software on the scientific computing platform of DESY, enabling them to obtain scientific results rapidly (Littlehales *et al.*, 2024 ▸). At the same time, this approach has allowed for debugging the software, testing its robustness and identifying further functionalities that need to be implemented in *MagStREXS*. In parallel to its development, *MagStREXS* has already contributed to several scientific cases (Simeth *et al.*, 2023 ▸; Rahn *et al.*, 2024 ▸; Moody *et al.*, 2025 ▸). The software is under continuous improvement to offer more functionalities, with the latest being the description of commensurate structures based on MSGs.

## Figures and Tables

**Figure 5:**
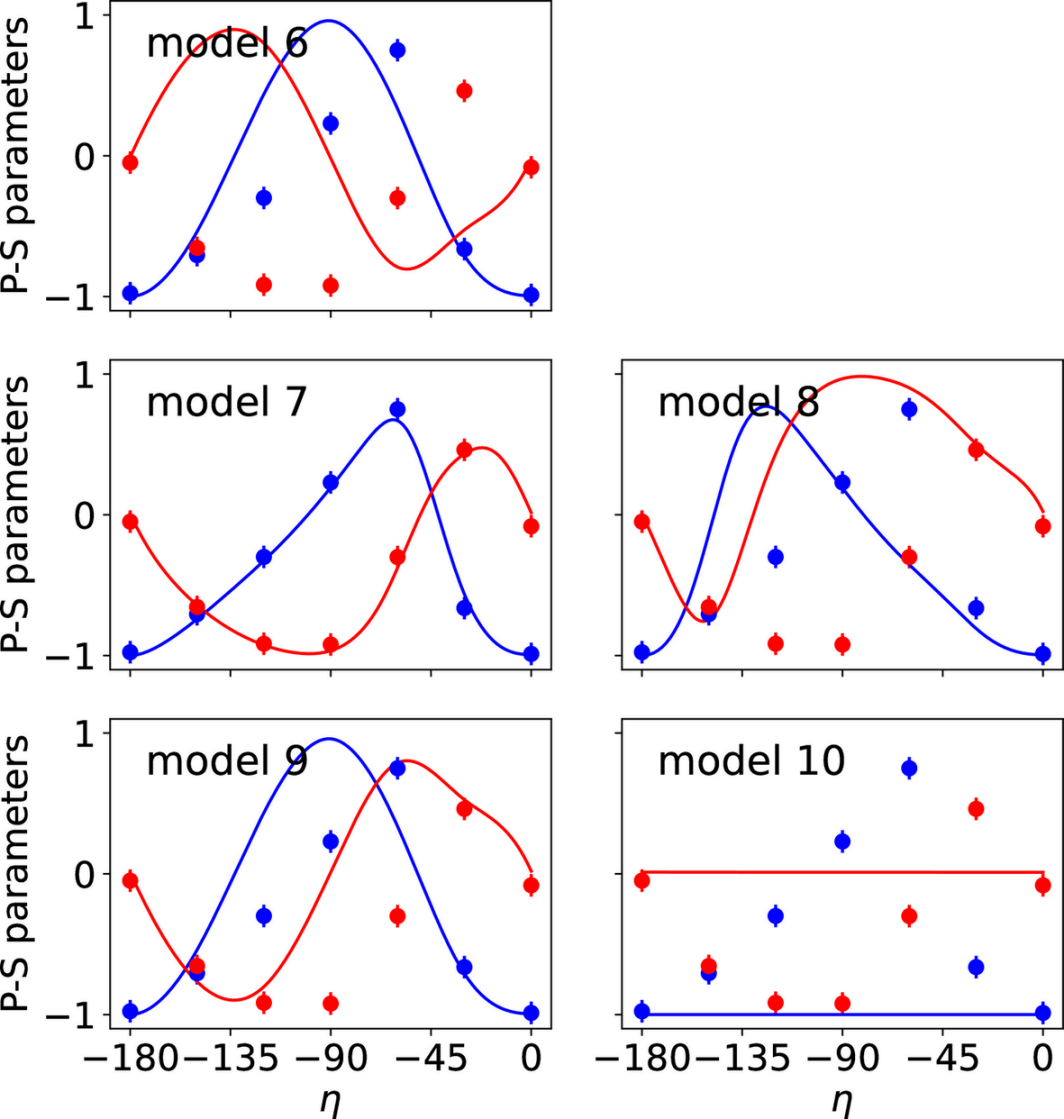
Comparison of observed (circles) and calculated (lines) P-S parameters *P*_1_ (blue) and *P*_2_ (red) corresponding to the reflection 005 for magnetic models given in Table 3 ▸. The P-S parameters vary with respect to the polarization of the incident X-ray beam (angle η). The best fit with the experimental P-S parameters corresponds to the values calculated for model 7.

**Figure 6:**
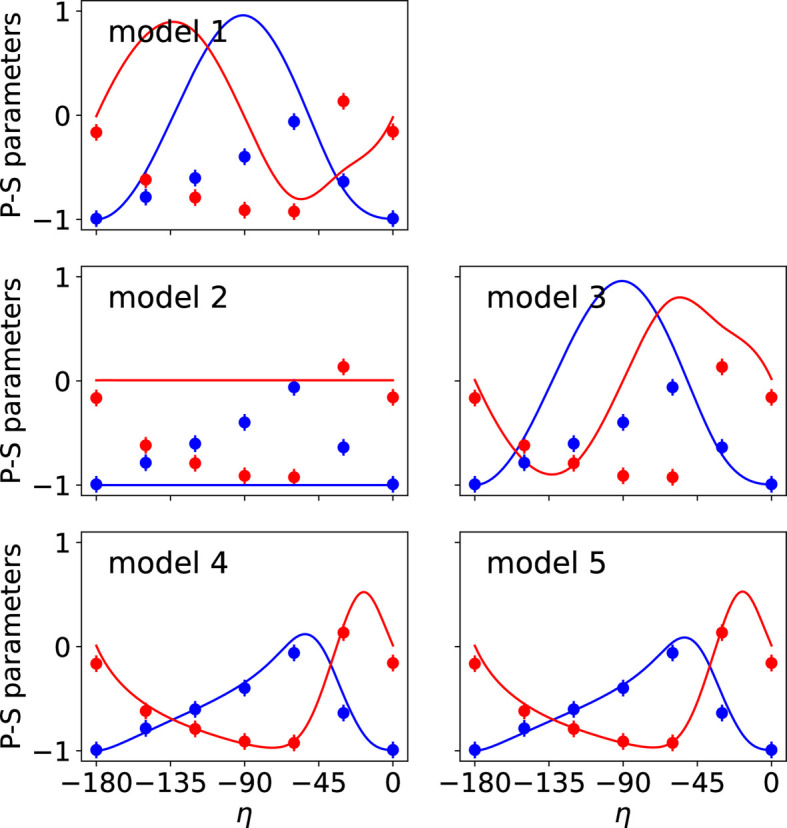
Observed and calculated P-S parameters *P*_1_ (blue) and *P*_2_ (red) corresponding to the reflection 009 for the magnetic models introduced in Section 6.1. The best fit with the experimental parameters corresponds to the values calculated for models 4 and 5.

**Figure 7:**
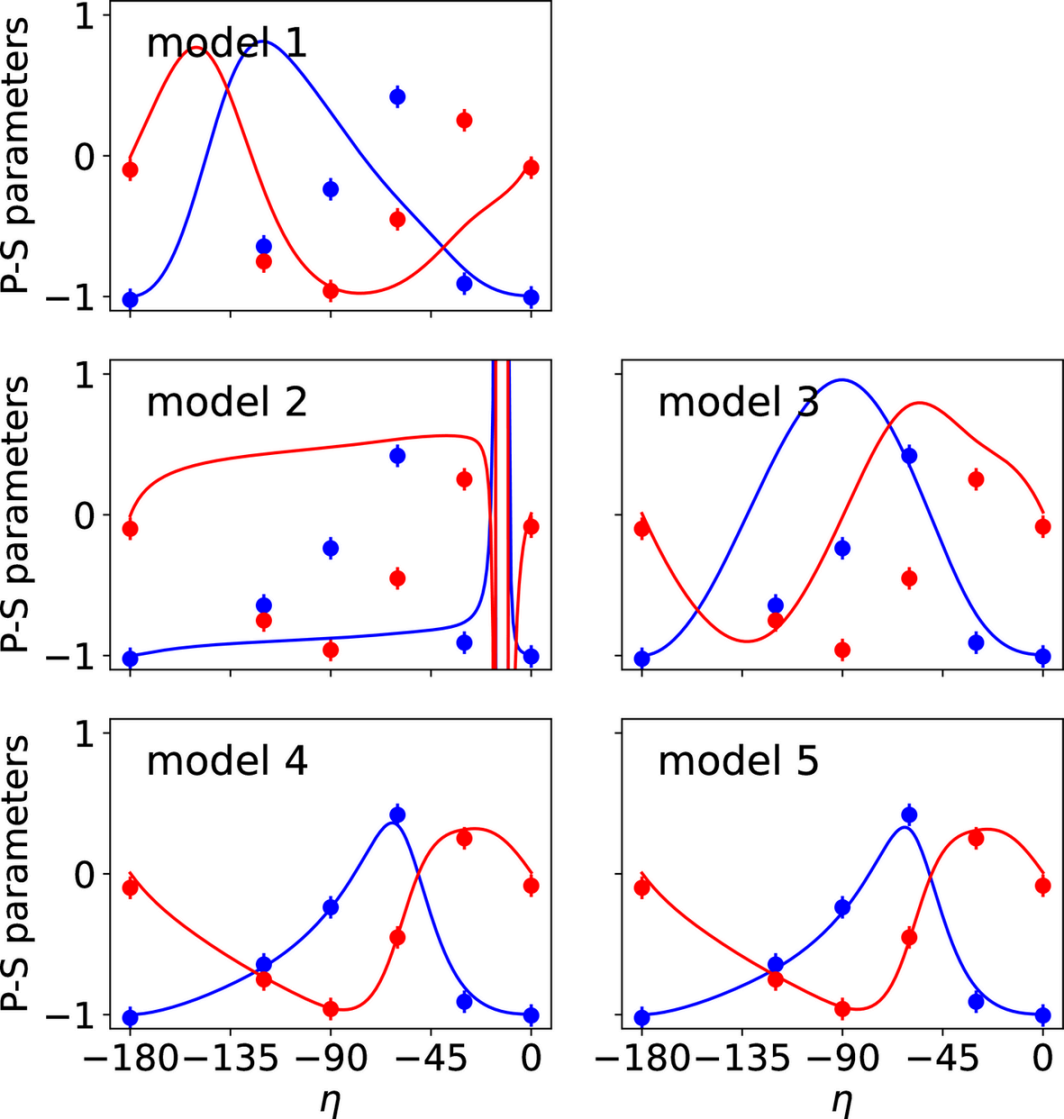
Observed and calculated P-S parameters *P*_1_ (blue) and *P*_2_ (red) corresponding to the reflection 016 for the magnetic models introduced in Section 6.1. The best fit with the experimental parameters corresponds to the values calculated for models 4 and 5.

**Figure 8:**
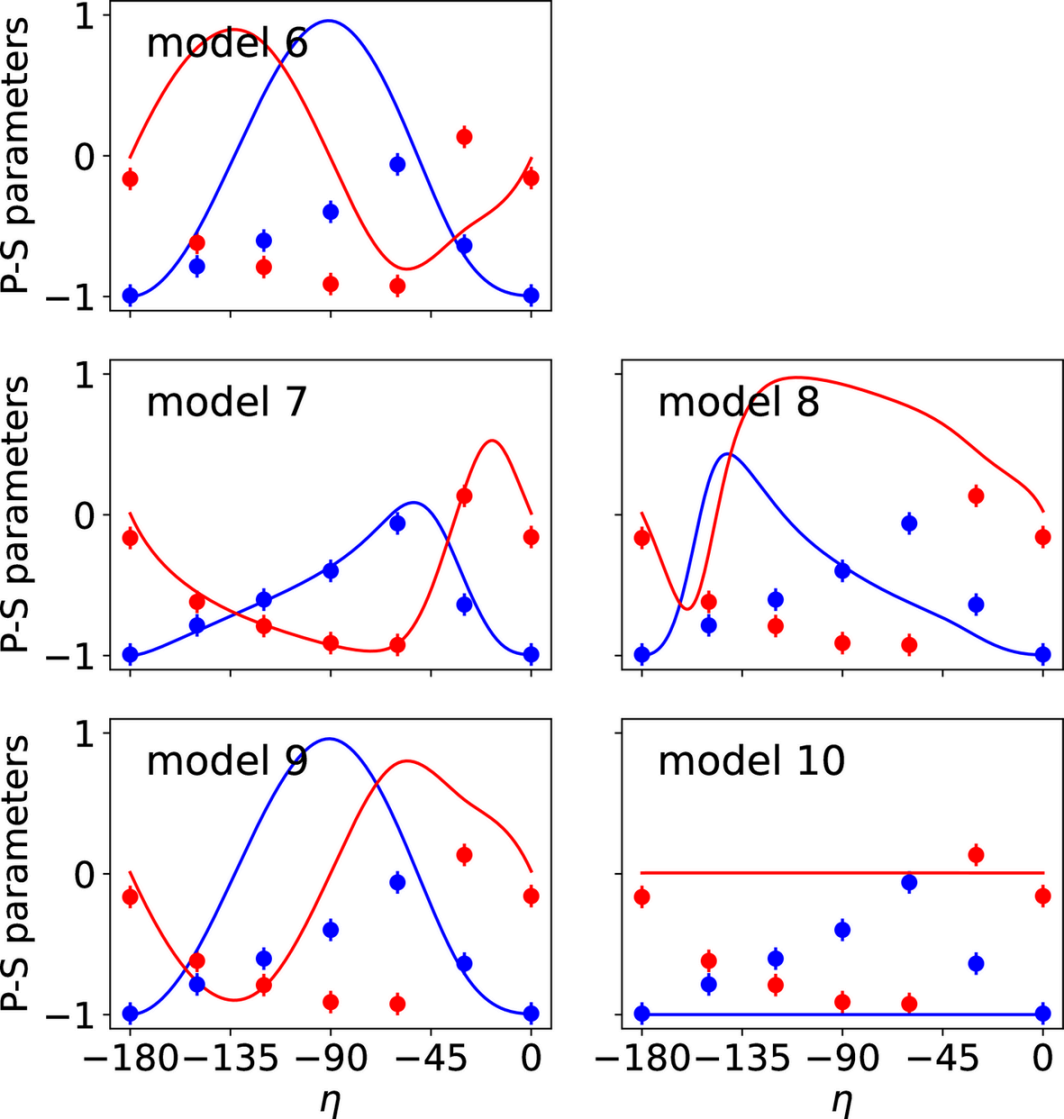
Observed and calculated P-S parameters *P*_1_ (blue) and *P*_2_ (red) corresponding to the reflection 009 for the magnetic models given in Table 3 ▸. The best fit with the experimental parameters corresponds to the values calculated for model 7.

**Figure 9:**
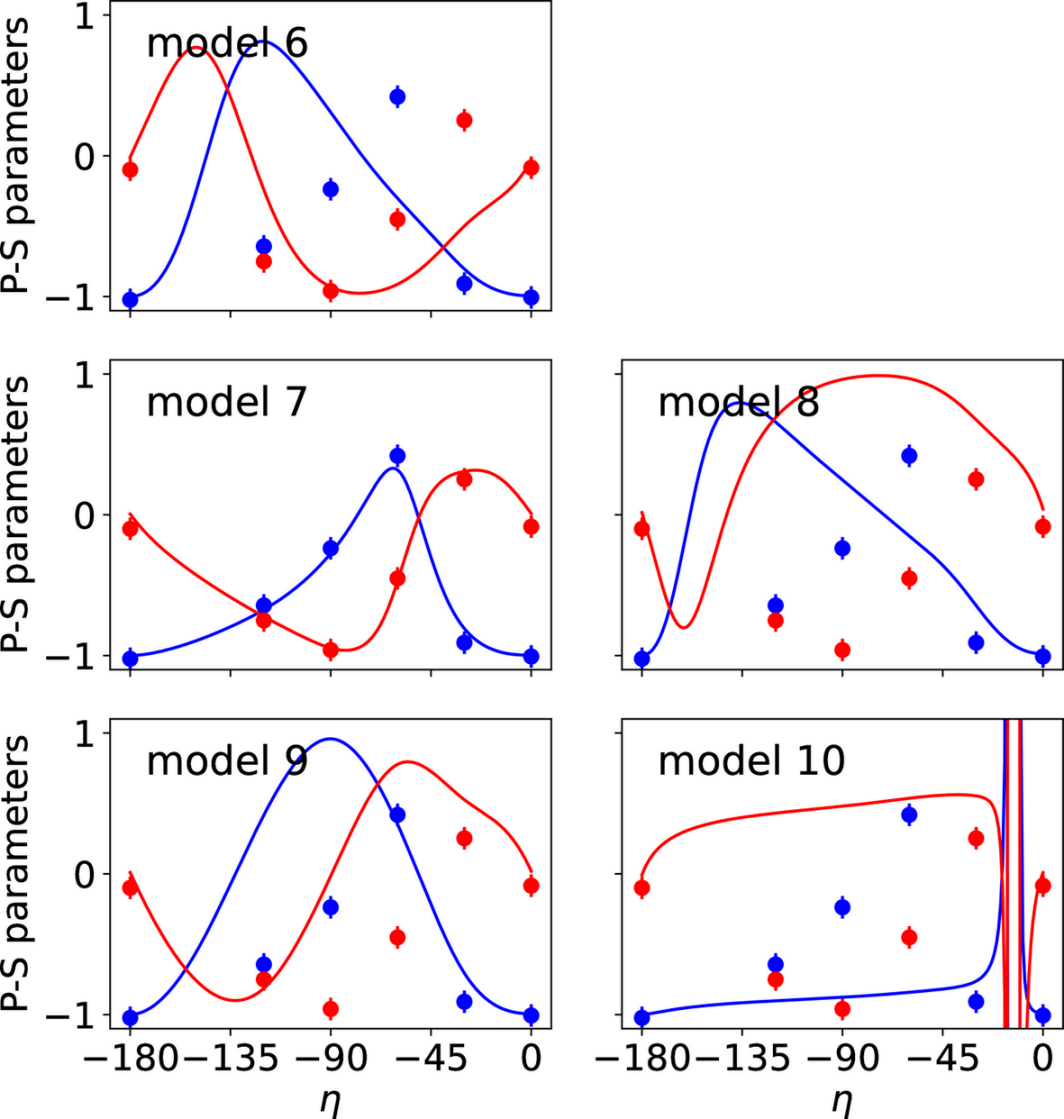
Observed and calculated P-S parameters *P*_1_ (blue) and *P*_2_ (red) corresponding to the reflection 016 for the magnetic models given in Table 3 ▸. The best fit with the experimental parameters corresponds to the values calculated for model 7.

**Table 1:** Basis vectors (BVs) associated with the irreps obtained in the representation analysis, where *m* and *im* correspond to the real and imaginary components, respectively, along the *a*, *b* and *c* axes

Irrep	BV	Atom	*m*_*a*_	*m*_*b*_	*m*_*c*_	*im*_*a*_	*im*_*b*_	*im*_*c*_
*mM*3+	ψ_1_	Eu	0	0	1	0	0	0
*mM*5+	ψ_2_	Eu	0	1	0	0	0	0
	ψ_3_	Eu	1	0	0	0	0	0

**Table 2:** Agreement factors obtained for the different models considered for the magnetic structure of EuNi_2_Ge_2_ The basis vectors corresponding to each magnetic model are indicated. Note that model 4 involves two independent mixing coefficients, while model 5 includes a constraint (*C*_3_ = −*C*_2_) that determines the orientation of the magnetic moments. Fitted values and standard deviations for the *C*_2_ and *C*_3_ parameters of model 4 are given in the text.

				*R*_PS_		
Model	Irrep	BV	*R*_B_	005	009	016
1	*mM*3+	ψ_1_	64.2	96.9	98.8	69.7
2	*mM*5+	ψ_2_	65.2	97.4	76.6	94.2
3	*mM*5+	ψ_3_	27.4	57.4	81.4	77.3
4	*mM*5+	*C*_2_ψ_2_ + *C*_3_ψ_3_	6.4	6.7	11.2	7.7
5	*mM*5+	*C*_2_ψ_2_ − *C*_2_ψ_3_	6.6	7.9	10.5	9.0

**Table 3:** Magnetic models obtained with the program *MAXMAGN* for the compound EuNi_2_Ge_2_ The transformation from the basis of the atomic structure to the standard setting of each MSG and the symmetry constraints on the magnetic moment for each model are also indicated.

Model	MSG	Transformation	Symmetry constraint
6	*P*_*I*_4/*mnc* (No. 128.410)	(**a**, **b**, **c**; 0, 0, 0)	(0, 0, *m*_*z*_)
7	*C*_*A*_*mca* (No. 64.480)	(**a** − **b**, **a** + **b**, **c**; 0, 0, 0)	(*m*_*x*_, −*m*_*x*_, 0)
8	*C*_*A*_*mca* (No. 64.480)	(**a** + **b**, −**a** + **b**, **c**; 0, 0, 0)	(*m*_*x*_, *m*_*x*_, 0)
9	*P*_*I*_*nnm* (No. 58.404)	(**c**, **b**, −**a**; 0, 0, 0)	(*m*_*x*_, 0, 0)
10	*P*_*I*_*nnm* (No. 58.404)	(**c**, −**a**, −**b**; 0, 0, 0)	(0, *m*_*y*_, 0)

**Table 4:** Agreement factors corresponding to the different models listed in Table 3 ▸

			*R*_PS_		
Model	MSG	*R*_B_	005	009	016
6	*P*_*I*_4/*mnc*	64.2	96.9	98.8	69.7
7	*C*_*A*_ *mca*	6.6	7.9	10.5	9.0
8	*C*_*A*_ *mca*	48.4	96.0	108.0	118.6
9	*P*_*I*_ *nnm*	27.3	57.4	81.4	77.3
10	*P*_*I*_ *nnm*	65.2	97.4	76.6	94.2

## Data Availability

All data supporting the plots within this paper and other results of this study are available upon reasonable request from the corresponding author.

## Appendix A Convention used in the expression of the magnetic moments

The negative sign in the Fourier series of equation (1) is consistent with the expression used in crystallography for the electron scattering density in terms of structure factors, written as

As a consequence, the REXS structure factors have a positive sign in the exponential factor. Adopting the standard solid-state physics convention would imply a negative sign in the exponential function, and the contributions associated with propagation vector **q**_1_ or with the combination **q**_1_ + **q**_2_ would appear at positions **H**_0_ − **q**_1_ and **H**_0_ − **q**_1_ − **q**_2_, respectively. The convention which we use is also consistent with the common formulation employed in the superspace formalism description as presented in a recent article on *JANA* by Henriques *et al.* (2024 ▸).

## Appendix B Antiferromagnetic phase of compound EuNi_2_Ge_2_: additional figures

Comparisons of observed and calculated P-S parameters corresponding to the reflections 009 and 016 are shown in Figs. 6 ▸–9 ▸ ▸ ▸. The figures include comparisons of the different models considered in Section 6. All the figures represent the variation in P-S parameters (*P*_1_ in blue and *P*_2_ in red) with respect to the polarization of the incident X-ray beam (angle η). The coloured circles and continuous lines represent the observed and calculated values, respectively.
